# GSDME介导的细胞焦亡在肺癌治疗中作用的研究进展

**DOI:** 10.3779/j.issn.1009-3419.2024.106.17

**Published:** 2024-07-20

**Authors:** Huan LI, Xudong TANG

**Affiliations:** ^1^524023 湛江，广东医科大学生物化学与分子生物学研究所，抗肿瘤活性物质研发协同创新中心; ^1^Institute of Biochemistry and Molecular Biology, Collaborative Innovation Center for Antitumor Active Substance Research and Development, Guangdong Medical University, Zhanjiang 524023, China; ^2^523808 东莞，广东医科大学医学技术学院; ^2^School of Medical Technology, Guangdong Medical University, Dongguan 523808, China

**Keywords:** 肺肿瘤, 成孔蛋白E, 细胞焦亡, Lung neoplasms, Gasdermin E, Pyroptosis

## Abstract

目前肺癌对人类健康构成了巨大威胁。尽管有关肺癌的治疗技术近年有了显著提高，但肺癌患者的5年生存率仍然不高。在此背景下，细胞焦亡（pyroptosis）这一独特的细胞死亡机制的发现，为探索肺癌治疗的新途径提供了全新的视角。特别是成孔蛋白E（gasdermin E, GSDME）在细胞焦亡过程中扮演的角色，提示其在肺癌治疗中具有巨大的应用潜力。近年来，GSDME介导的细胞焦亡在肺癌生长、肺癌微环境中的调节作用及GSDME甲基化对肺癌治疗影响的研究取得了较大的进展，本文对这些研究进展进行了总结，并对GSDME介导的细胞焦亡在肺癌治疗中的潜力及潜在的副作用进行了分析，旨在为开发更有效的肺癌治疗策略提供理论基础。

## Abstract (en)

Lung cancer causes a significant threat to human health. Despite considerable advancements in the treatment technologies in recent years, the five-year survival rate for lung cancer patients remains low. In this context, the discovery of pyroptosis, a unique cell death mechanism, offers a novel perspective for exploring new pathways of lung cancer treatment. Particularly, the role of gasdermin E (GSDME) in the process of pyroptosis reveals its tremendous potential in lung cancer therapy. Recent studies have made considerable progress in understanding the role of GSDME-mediated pyroptosis in lung cancer growth, the lung cancer microenvironment, and the effect of GSDME methylation on lung cancer treatment. This paper summarizes these research advancements and analyzes the potential and possible side effects of GSDME-mediated pyroptosis in lung cancer therapy, aiming to provide a theoretical foundation for developing more effective strategies for lung cancer treatment.

肺癌不仅是全球最常见的恶性肿瘤之一，还是导致癌症死亡的主要原因^[1]^。尽管近年来在早期诊断和治疗方法方面取得了一些进展，但肺癌的5年生存率仍然较差。因此，探求肺癌有效预防策略和新治疗方法越来越受到重视。

细胞焦亡是一种由成孔蛋白家族（gasdermin, GSDM）介导的炎症性程序性细胞死亡方式^[2]^。GSDM家族成员包括gasdermin A（GSDMA）、gasdermin B（GSDMB）、gasdermin C（GSDMC）、gasdermin D（GSDMD）、gasdermin E（GSDME）及pejvakin（PJVK），该家族具有共同的结构特征，包括氨基末端结构域（N-terminal domain, NTD）和羧基末端结构域（C-terminal domain, CTD）。早期研究^[3,4]^认为GSDM介导的细胞焦亡主要有两条途径：（1）经典炎症小体途径：由半胱天冬氨酸蛋白酶-1（caspase-1）介导，通过模式识别受体识别病原体激活炎症小体，切割GSDMD形成膜孔；（2）非经典炎症小体途径：由caspase-4/5/11介导，通过细菌脂多糖直接激活，切割GSDMD形成膜孔^[4]^。近年来又发现凋亡相关caspase-3介导的细胞焦亡途径^[5]^，其通过激活caspase-3从而切割GSDME^[6]^，最终介导焦亡的发生。

GSDME，亦称作DFNA5（deafness autosomal dominant 5），是一种由人类基因编码的蛋白质，属于GSDM蛋白家族^[7]^。最初发现GSDME与遗传性耳聋有关，近年来的研究^[8]^发现GSDME具有促进细胞发生焦亡的作用，但其激活机制与GSDMD等其他家族成员不同^[9,10]^。本文即对近年来GSDME介导的细胞焦亡在肺癌生长、肺癌微环境中的作用及GSDME甲基化对肺癌治疗影响的研究进展进行总结，并对GSDME介导的焦亡在肺癌治疗中的潜力和潜在副作用进行分析。

## 1 GSDME介导的细胞焦亡

GSDME介导的细胞焦亡主要依赖于caspase-3的激活^[5]^，相比于其他焦亡途径所依赖的caspase-1或caspase-4/5/11^[3,4]^，研究^[6]^发现在GSDME介导的细胞焦亡过程中，细胞程序性死亡信号（如细胞凋亡信号）激活使caspase-3可以裂解GSDME蛋白，从而使CTD与NTD分离，NTD迁移到细胞膜上聚合形成大孔洞，增强细胞膜的渗透性^[11]^，导致细胞内外电解质失衡，细胞吸水膨胀并最终死亡^[2]^（图1）。上游信号在生理和病理条件下有所不同^[12,13]^：在生理条件下，细胞凋亡信号（如DNA损伤、细胞应激）通过激活caspase-3切割GSDME，引发焦亡；在病理条件下，肿瘤微环境中的应激反应、化疗药物诱导和免疫反应等可通过激活caspase-3切割GSDME，导致肿瘤细胞焦亡。

**图1:**
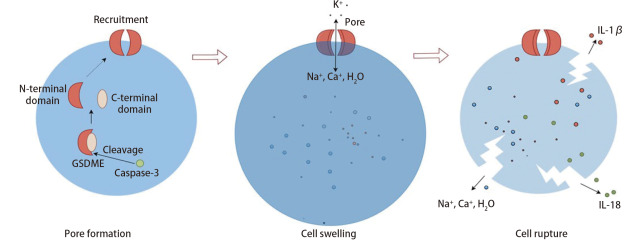
GSDME介导的细胞焦亡机制图

GSDME介导的焦亡不仅导致细胞死亡，还会释放细胞内的炎症因子白介素-1β（interleukin-1β, IL-1β）和IL-18，吸引免疫细胞（如CD8^+ ^T细胞等）进入肿瘤组织，从而增强抗肿瘤免疫^[14,15]^（图1）。提示细胞焦亡与免疫系统密切关联，显示出GSDME介导的细胞焦亡在调节肺癌等肿瘤微环境中的免疫细胞动态及对肺癌细胞生长和治疗的作用。因此，GSDME在调控肺癌等肿瘤细胞的死亡以及影响肿瘤免疫环境方面具有独特的生物学意义。

## 2 GSDME介导的细胞焦亡在肺癌治疗中的作用

### 2.1 GSDME介导的细胞焦亡对肺癌生长的影响

研究^[16,17]^发现，GSDME在小细胞肺癌（small cell lung cancer, SCLC）和非小细胞肺癌（non-small cell lung cancer, NSCLC）中的表达水平显著增加，高表达GSDME的肺癌患者显示出较好的临床特征，包括较低的淋巴结转移率、较长的术后生存期、较好的预后和较少的肿瘤复发。这种高表达水平使得caspase-3可通过裂解GSDME而介导肺癌细胞发生焦亡，不仅能促使肺癌细胞死亡，还能释放免疫刺激因子，进一步增强抗肿瘤免疫反应^[18,19]^。值得注意的是，GSDME的表达水平可能因受到其他因素的影响而降低，主要影响因素包括表观遗传修饰和蛋白功能丧失等。有研究^[20]^指出，肺癌细胞可通过DNA甲基化等表观遗传修饰抑制GSDME的表达。此外，GSDME蛋白可能发生突变，导致其功能丧失，进一步避免了焦亡的发生，从而使癌细胞逃避免疫系统的清除^[21]^。

有报道^[19,22,23]^发现，顺铂和紫杉醇等化疗药物在GSDME高表达时激活caspase-3，继而切割GSDME，使得原本的凋亡过程转变为焦亡，从而抑制肺癌的生长。此外，顺铂对GSDME高表达的肺癌细胞的疗效优于紫杉醇^[23]^，其通过激活caspase-3/GSDME途径，能有效诱导A549细胞发生焦亡，显著抑制其生长。Peng等^[24]^的研究发现，顺铂能在GSDME高表达的A549细胞中诱导焦亡，触发抗肿瘤免疫细胞的浸润，增强其抗肿瘤效应。因此，GSDME可通过介导焦亡增强顺铂对NSCLC的抗肿瘤作用。

CC-115是一种用于I期临床试验的新型抗肿瘤化合物。研究^[25]^发现，这种化疗药物通过诱导GSDME介导的焦亡来抑制肺腺癌的生长，其机制是通过抑制Akt磷酸化，削弱其对Bax的抑制作用，从而通过Bax-线粒体内在途径诱导细胞焦亡。GSDME的激活和随后发生的焦亡可能是癌细胞对这种治疗的反应机制。程序性细胞死亡蛋白配体1（programmed cell death ligand 1, PD-L1）抑制剂是一类针对PD-L1的免疫检查点抑制剂，可有效阻断肿瘤免疫逃避途径^[26]^。有研究^[27]^认为，顺铂诱导的GSDME介导的细胞焦亡可以增强PD-L1抑制剂的疗效。因此，顺铂诱导的细胞焦亡与PD-L1抑制剂的结合，可能是治疗SCLC的有效策略^[18,28]^。这些研究说明GSDME介导的焦亡在肺癌治疗中具有重要作用，提示其可作为肺癌的潜在治疗策略。

### 2.2 GSDME在肺癌微环境中的调节作用

肺癌微环境包括基质、血管和多种免疫细胞，其对肺癌的发展和治疗具有较大的影响^[29]^。肺癌微环境的细胞相互作用、血供状况以及免疫应答不仅影响肺癌的生长和转移，还会影响肺癌对治疗的反应^[29]^。研究肺癌微环境有助于深入了解肺癌发生发展的分子机制，从而开发更有效的疗法。

研究^[30]^表明，GSDME在肺癌细胞中的激活不仅通过诱导焦亡促进局部炎症反应，还能显著调节肺癌微环境。焦亡作为一种促炎症性细胞死亡方式，可通过释放细胞内容物和炎症介质，有效激活肺癌微环境中的免疫细胞^[31]^。这不仅为免疫细胞识别和攻击肺癌细胞提供信号，还能改变肺癌微环境中免疫细胞的组成和功能，如明显增强抗肿瘤CD8^+ ^T细胞的活性和促进巨噬细胞的吞噬作用^[24]^。通过这种方式，GSDME介导的焦亡有助于增强对肺癌细胞的免疫反应，提升免疫系统清除肺癌细胞的能力。Hu等^[32]^发现，GSDME可调节肺癌微环境的免疫状态和细胞信号转导如核因子-κB（nuclear factor-κB, NF-κB）和干扰素基因刺激因子（stimulator of interferon genes, STING）通路，从而影响肺癌等肿瘤的生长、转移和对治疗的反应。

综上所述，GSDME可通过调控GSDME的活性来影响肺癌的免疫环境，改善肺癌微环境，从而达到治疗肺癌的目的。

### 2.3 GSDME甲基化对肺癌治疗的影响

GSDME甲基化是一种表观遗传修饰，能够影响GSDME基因的表达和功能^[33]^。GSDME基因的甲基化通常发生在其启动子区域，作为基因表达调控的关键区域，启动子区域含有大量的CpG岛，而CpG岛的甲基化状态可以直接影响基因的转录活性^[34,35]^。在GSDME基因中，当启动子区域的CpG岛高度甲基化时，通常会导致基因表达的下降，这是因为甲基化会阻止转录因子等调控蛋白与DNA结合，从而抑制基因的转录^[20,34,36]^。有研究^[18]^报道，GSDME在肺癌中表达降低，GSDME的高甲基化可能是其主要原因^[34]^。GSDME表达降低与肺癌患者的不良预后密切相关，肺癌通常表现出更高的侵袭性和转移性，导致患者的生存率显著降低^[37]^。此外，由于GSDME介导的焦亡路径被抑制，肺癌可能对化疗药物和免疫治疗产生抵抗性^[19]^。

多项研究^[38,39]^已证明，GSDME甲基化对肿瘤的发生发展有重要影响。Hu等^[40]^发现，GSDME的甲基化水平与肺癌的进展和转移密切相关，肺癌细胞中GSDME的高甲基化水平直接影响了GSDME的正常表达。去甲基化药物如5-氮胞苷和地西他滨通过抑制DNA甲基转移酶的活性，能有效去除甲基化修饰，恢复GSDME的表达，诱导细胞焦亡，增强抗肿瘤免疫反应^[41,42]^。一些临床试验正在评估去甲基化药物与其他免疫治疗联合使用的效果，以期提升肺癌患者的生存率和生活质量。

总之，GSDME的甲基化在肺癌中具有重要的生物学意义，其甲基化状态和表达水平可以作为肺癌的诊断和预后标志物。然而，对这一领域的全面理解还需要更多的研究，进一步探究GSDME基因的甲基化机制及其在肺癌发生发展中的具体作用，有助于肺癌的诊断和治疗。

## 3 GSDME的激活在肺癌治疗中的应用潜力与潜在的副作用

相对于传统化疗，靶向治疗在肺癌治疗中有较多优势，包括更具针对性、较小的副作用、较高的生存率、治疗个性化等^[43]^。在肺癌治疗中，分子靶向疗法不但能诱导细胞发生凋亡，而且可以激活GSDME依赖的细胞焦亡^[16]^，从而抑制肺癌细胞的生长，提示针对GSDME依赖的细胞焦亡的分子靶向治疗可能是增强肺癌治疗效果的一种有前景的策略。

研究^[25,44]^表明，激活或增强GSDME的功能，可促进肺癌细胞的焦亡，从而抑制肺癌生长。未来，随着对GSDME相关途径的深入理解和药物递送技术的发展，通过激活GSDME依赖的焦亡的疗法有望成为肺癌治疗的重要手段，为患者提供更有效的治疗选择。针对GSDME的药物研发主要集中在两个方面：一是寻找能直接触发GSDME活化的化学物质；二是结合已有的化疗药物，通过调控GSDME的表达或活性来增强药物的疗效。值得一提的是，GSDME也可被自然杀伤（natural killer, NK）细胞释放的颗粒酶B直接切割后激活，增强肿瘤浸润免疫细胞的功能，发挥抗肿瘤活性^[19,45]^。

GSDME介导细胞焦亡治疗肺癌可为患者提供基于个体GSDME表达和活性的个性化治疗方案，其优点包括增强化疗效果、激活免疫系统进而有效清除肿瘤，其研发不仅有望改善肺癌患者的治疗效果和生活质量，还可通过与其他治疗手段结合，进一步提高肺癌治疗的有效性。未来的研究将集中在优化这些药物与其他治疗方法的协同作用，以及探索其在不同类型和阶段肺癌中的应用。

尽管GSDME介导的细胞焦亡在肺癌治疗中展示了良好的发展潜力，但其可能伴随一定的副作用和安全性问题：（1）非特异性组织损伤：GSDME介导的细胞焦亡在特异性组织损伤方面可能不仅限于目标病变组织，还可能影响周围健康细胞，从而在癌症治疗中引发炎症和组织坏死^[46]^；（2）炎症反应的加剧：GSDME的活化会导致细胞膜破裂，释放炎症介质如IL-1β等，引起局部或全身的炎症反应，这种过度的炎症可能导致组织损伤、自身免疫反应或其他严重的炎症相关疾病^[47]^；（3）免疫系统的潜在影响：GSDME介导的细胞焦亡可通过激活免疫系统影响免疫细胞的功能，但这种影响既有利于增强抗肿瘤免疫反应，也可能导致免疫系统失调^[48]^；（4）长期效果和慢性问题：长期激活GSDME可能导致持续的炎症状态和功能障碍，从而引起慢性健康问题^[34]^。

为解决这些潜在的副作用与安全性问题，开发基于激活GSDME的疗法时应考虑精确递送系统、剂量控制、临床监测和组合治疗。这些策略有助于最大优化基于GSDME依赖的细胞焦亡疗法的疗效，同时降低潜在的副作用和安全风险。

## 4 结语与展望

GSDME介导的细胞焦亡在肺癌治疗中的研究进展为我们提供了新的视角和潜在的治疗策略。细胞焦亡作为一种独特的细胞死亡方式，其在肺癌中的作用逐渐受到重视。GSDME作为这一过程中的关键分子，对其进行深入研究不仅有助于理解肺癌的发生发展机制，同时也为开发新的肺癌治疗方法提供了可能。尽管如此，目前的研究还存在一些不足之处：（1）机制不完全明确：GSDME介导的细胞焦亡在肺癌中的具体分子机制尚不完全清楚，需要进一步的分子层面研究；（2）临床应用限制：GSDME的激活与调控在临床上的应用仍面临诸多挑战，包括如何精确控制其活性，以及如何将这一过程应用于具体的治疗方案中；（3）副作用与安全性^[34,47]^：GSDME激活可能引起的副作用和长期安全性问题是制约GSDME介导的细胞焦亡策略应用于临床肺癌治疗的关键因素；（4）GSDME的表达水平：GSDME在肺癌中的表达水平存在争议，需要进一步研究以揭示GSDME在不同肺癌类型中的表达模式及其生物学意义，从而准确解释这些差异。

总之，GSDME介导的细胞焦亡在肺癌防治中的作用取得了显著进展，展示出了良好的应用前景。然而，这一领域仍需进一步研究，尤其是需要更多的临床试验来验证GSDME介导的细胞焦亡在肺癌治疗中的实际效果和安全性。随着对GSDME的深入了解，有望开发出更加有效和安全的肺癌治疗方法，从而改善患者的生存率和预后。
